# Cancer Drug Price and Novelty in Mechanism of Action

**DOI:** 10.1001/jamanetworkopen.2023.47006

**Published:** 2023-12-11

**Authors:** Miloš D. Miljković, Jordan Tuia, Timothée Olivier, Alyson Haslam, Vinay Prasad

**Affiliations:** 1 Cartesian Therapeutics, Gaithersburg, Maryland; 2 Department of Epidemiology and Biostatistics, University of California, San Francisco; 3 Department of Oncology, Geneva University Hospital, Geneva, Switzerland; 4 Department of Medicine, University of California, San Francisco

## Abstract

**Importance:**

Many economic theories point to regulatory issues and subsidization of research and development costs as the primary factor in the high cancer drug prices in the US. Even so, the association between the median annual cost and novelty of cancer drugs approved in the US remains unclear.

**Objective:**

To evaluate the association between the median annual cost and novelty of cancer drugs approved in the US over a 6-year period.

**Design, Setting, and Participants:**

This cross-sectional study included all cancer drugs approved by the US Food and Drug Administration (FDA) from January 1, 2015, to December 31, 2020. Drug names, indications, manufacturer, dosage, and measures of activity/efficacy were extracted from the FDA announcement. The search was performed in December 2021. Data were analyzed from January 2022 until April 2022.

**Main Outcomes and Measures:**

Annual cost of treatment was calculated based on average wholesale price collected from the 2021 Micromedex Red Book database. Mechanism of action was inferred from trial publication or its references.

**Results:**

There were 224 cancer drug approvals across 119 individual drugs, with a median annual cost of $196 000 (IQR, $170 000-$277 000). Gene and viral therapies were the most expensive (median, $448 000 [IQR, $448 000-$479 000]), followed by small molecule therapy (median, $244 000 [IQR, $203 000-$321 000), and biologics (median, $185 000 [IQR, $148 000-$195 000]). There was no significant difference in cost between first-in-class, next-in-class, and subsequent approvals of an already approved drug.

**Conclusions and Relevance:**

Findings of this study indicate that the median annual price of anticancer drugs in the US is not associated with the novelty of their mechanism of action.

## Key Points

**Question:**

Is the median annual cost of cancer drugs associated with the novelty of cancer drugs approved in the US over a 6-year period?

**Findings:**

In this cross-sectional study, between January 1, 2015, and December 31, 2020, there were 224 cancer drug approvals across 119 individual drugs. The median annual cost was $196 000, and there was no significant difference in cost between first-in-class, next-in-class, and subsequent approvals of an already approved drug.

**Meaning:**

The results of this study suggest that drug pricing cannot be explained by innovations alone; further study is warranted.

## Teaser

This cross-sectional study assesses US Food and Drug Administration anticancer drug approvals over 6 years in terms of cost and novelty in mechanism of action.

## Introduction

As cancer drug prices in the US continue to increase, the reason for the increase continues to be a core and inflammatory topic in medical policy. Many economic theories point to regulatory issues as the primary factor.^1^ However, a common defense for the high prices has been the US subsidization of global drug research and development, the costs of which are then passed on to US citizens in the form of drug prices. This framework reportedly explains why the US prices are double those in Europe.^2^ This perception that research and development are dependent on current drug prices is so common that the American Society of Clinical Oncology, in its position statement on addressing the affordability of cancer drugs, notes the need to balance the financial toll of cancer care with preserving innovation.^3^

Some evidence however complicates this statement. An analysis by Yu et al^4^ suggests that excess spending by the US exceeds the cumulative global research and development (R&D) portfolio of all drug companies. In other words, even if the US subsidized all of the world’s R&D, its citizens pay even more. A study from 2017^5^ questioned the cost of R&D and suggested that lower figures than a widely-publicized 2.6 billion dollar estimate to bring 1 drug to market. More specifically, the price to bring a drug to market was estimated to be higher for drugs with a novel mechanism of action compared with next-in-class drugs.^5^

Looking at the prevalence of such innovation in anticancer drugs, a previous study^6^ found 332 new drug approvals over 12 years, with 53 (16%) having a novel mechanism of action, 84 (25%) being first approval of a next in class compound, and 195 (59%) being subsequent approvals of the same drug. A similar study^7^ also found drug approvals from 2015 to 2020 showing weak correlation between clinical benefit and pricing.

The increasing cost of anticancer drugs is notable, and the many factors associated with the phenomenon are currently being clarified in the literature. With the constant justification of innovation as a defining aspect of the market, we wanted to explore innovation further. Specifically, we sought to describe these temporal trends by estimating US Food and Drug Administration (FDA) anticancer approvals and evaluate cost and novelty in mechanism of action.

## Methods

### Data Set

This was a retrospective cross-sectional analysis of all cancer drugs approved by the FDA from January 1, 2015, to December 31, 2020, assembling metrics of their activity or efficacy, and their annual cost or course of treatment as previously described.^6,8^ We adhered to the Strengthening the Reporting of Observational Studies in Epidemiology (STROBE) reporting guideline. The search was performed in December 2021. Data were analyzed from January 2022 until April 2022. Because we used publicly available data, and this was not human participant research in accordance with 45 CFR §46.102(f), we did not submit this study to an institutional review board or require informed consent procedures.

### Search Strategy

All generic drug names were extracted from the FDA website notifications of oncology drug approvals in the stated period. Drugs approved for palliative or symptomatic care, biosimilars, alternative dosage, new mode of administration, for indications limited to pediatric populations, or updated clinical efficacy for the same indication (eg, trametinib and dabrafenib received accelerated approval for unresectable or metastatic melanoma with BRAF V600E or V600K mutations in 2014 but was granted prior approval in 2015 with updated clinical trial results for labeling) were excluded.

### Data Abstraction

Data extracted from the FDA announcement and label information included date of approval, drug names, indication, manufacturer, recommended dosage, and measures of efficacy. The total number of drugs from each manufacturer was used to categorize manufacturers into quartiles.

Mechanism of action was inferred from the trial publication or its references, and a previous study.^6^ Coding of mechanism of action and novelty classification per approval has been previously described.^6^ We classified each approval into 3 categories: first approval of a new mechanism of action compound, next-in-class approval regardless the tumor type, and subsequent approval of the same drug.^6^ Drugs were additionally grouped according to their mechanism of action into small molecules (including kinase inhibitors and immunomodulatory drugs), biologics (including monoclonal and bispecific antibodies, antibody-drug conjugates, and fusion proteins), cytotoxic drugs (including cytotoxic chemotherapy and radio-conjugates), gene and oncolytic virus therapy, and hormonal therapy.

The average wholesale price for each drug was collected from the 2021 Micromedex Red Book (IBM) database. Cost was determined by calculating the recommended dose in yearly course of treatment by the average wholesale price. Dosage requirements were calculated for adults weighing 60 kg. When appropriate, doses were rounded up to account for unused product.

As a surrogate for total federal research funding in each disease area we used the National Cancer Institute Budget Fact Book.^9^ Data from years 2015 to 2020 were aggregated and the 5 most funded tumor types were categorized as high funding while the rest were categorized as low.

### Statistical Analysis

Descriptive statistics were calculated and presented as numbers and percentages for categorical variables and medians and IQRs for continuous variables, with prices rounded to the nearest thousand. For categorical variables, a Kruskal-Wallis test was used to determine global differences between median annual cost of subgroups. The results were considered statistically significant at *P* < .05 using a 2-tailed test. For significant results, a pairwise Wilcoxon rank sum test was used for narrowing. No outliers were excluded for analysis. The analysis was conducted in R version 4.1.2 (R Foundation for Statistical Computing) and cross-checked in Mathematica version 12.3.1.0 (Wolfram).

## Results

We identified 273 drugs in an initial review of FDA oncologic drug approvals. After applying exclusion criteria, 224 cancer drug approvals across 119 individual drugs were analyzed. Across all tumor types, median annual cost for a course of an anticancer drug was $196 000 (IQR, $170 000-$277 000) (Table).

**Table.** Characteristics of US Food and Drug Administration Drug Approvals (2015-2020) by Novelty Across all Tumors

Characteristic	Approvals, No. (%)^a^		
	Approved based on a new mechanism of action (n = 29)	First approvals of a next-in-class drug (n = 55)	Subsequent approvals of the same drug (n = 140)
Annual cost, median (IQR), $	$292 175 ($170 457-$376 512)	$206 464 ($156 372-$273 529)	$190 393 ($175 995-$250 942)
Novelty of drug per indication			
Approved based on a new mechanism of action in a new tumor type	28 (97)	10 (18)	43 (31)
First approvals within 1 tumor type of a next-in-class drug	1 (3.4)	43 (78)	25 (18)
Subsequent approvals of the same drug in the same tumor type	0	2 (3.6)	72 (51)
Target			
Other	27 (93)	38 (69)	64 (46)
PD1	0	1 (1.8)	44 (31)
PD-L1	1 (3.4)	2 (3.6)	13 (9.3)
PARP	0	3 (5.5)	10 (7.1)
Pan-kinase	0	4 (7.3)	7 (5.0)
Cell cycle	1 (3.4)	7 (13)	2 (1.4)
Tumor type			
Other	18 (62)	28 (51)	88 (63)
NSCLC	1 (3.4)	8 (15)	23 (16)
Breast	2 (6.9)	10 (18)	9 (6.4)
Myeloma	4 (14)	3 (5.5)	7 (5.0)
AML	3 (10)	4 (7.3)	5 (3.6)
Melanoma	1 (3.4)	2 (3.6)	8 (5.7)
Mechanism			
Small molecule therapy	12 (41)	34 (62)	56 (40)
Biologics	11 (38)	11 (20)	77 (55)
Cytotoxic therapy	4 (14)	5 (9.1)	2 (1.4)
Hormonal therapy	0	3 (5.5)	4 (2.9)
Gene and oncolytic virus therapy	2 (6.9)	2 (3.6)	1 (0.7)

Abbreviations: AML, acute myeloid leukemia: NSCLC, non–small cell lung cancer; PARP, P, poly (ADP-ribose) polymerase; PD1, programmed death 1; PD-L1, programmed death ligand 1.

^a^ The median approval date for approved based on a new mechanism of action was November 11, 2018, for first approvals of a next-in-class drug was August 16, 2018, and for subsequent approvals of the same drug was July 10, 2018.

Prices varied significantly between drugs by mechanism of action. Gene and virus therapy had the highest median annual cost ($448 000; IQR, $448 000-$479 000; n = 5; *P* = .048 compared with all others), followed by small molecule therapy ($244 000; IQR, $203 000-$321 000; n = 100; *P* < .001 compared with all others), biologics ($185 000; IQR, $148 000-$195 000; n = 100; *P* < .001), cytotoxic therapy ($183 000; IQR, $108 000-$206 000; n = 12), and hormonal therapy ($177 000; IQR, $132 000-$185 000; n = 7) (Figure, A).

**Figure.**
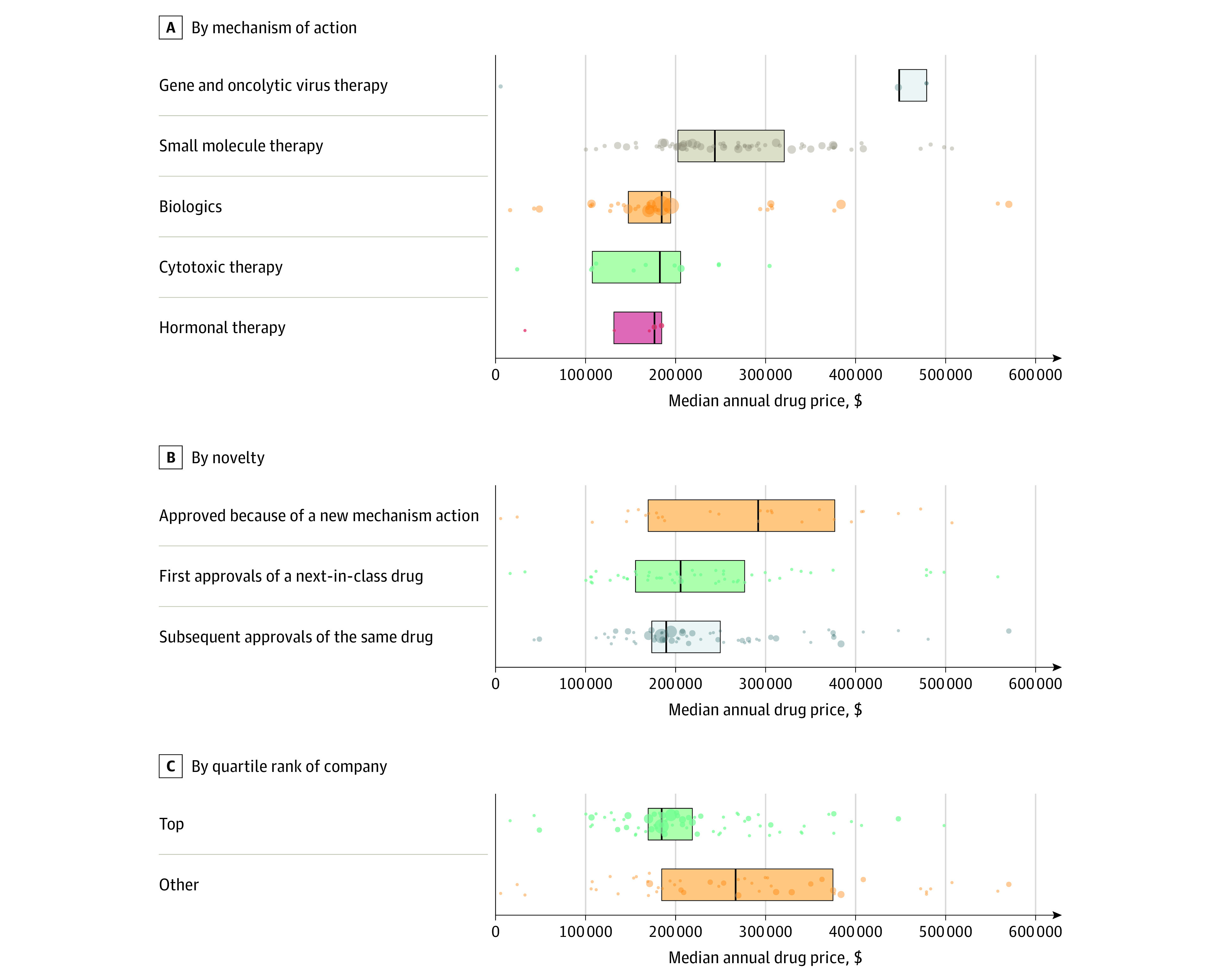
Median Annual Costs of Drugs by Mechanism of Action, Novelty, and Quartile Rank (2015-2020) A. Annual cost of anticancer drugs by mechanism of action for US Food and Drug Administration approved drugs (2015-2020). B. Annual cost of drugs based on novelty across all tumors for US Food and Drug Administration approved drugs (2015-2020). C. Annual cost of drugs by approval per company for US Food and Drug Administration approved drugs (2015-2020), stratified by number of drug approvals per company (top quartile vs other). Outliers were excluded from the figure for clarity. Each data point represents a unique drug within the novelty group. Size represents number of approvals.

There were no significant differences in median annual cost between novel drugs ($292 000; IQR, $170 000-$377 000; n = 29), next-in-class drugs ($206 000; IQR, $156 000-$277 000; n = 55; *P* = .14 compared with novel drugs), and subsequent approvals of an already approved drug ($190 000; IQR, $174 000-$250 000; n = 140; *P* = .09 compared with novel drugs) (Figure, B). Of the distinct drugs with a debut approval in our study time period, 9 of the 29 first-in-class and 17 of the 57 next-in-class drugs had at least 1 subsequent approval thereafter (eTable in Supplement 1).

Of the 37 manufacturers, the top quartile of drug manufacturers in number of drugs approved were responsible for 67% (150 of 224) of the drug approvals. The median annual cost of drugs from the top quartile of manufacturers was significantly lower than the cost of drugs from the bottom 3 quartiles ($185 000; IQR, $170 000-$219 000 for top quartile vs $267 000; IQR, $186 000-$375 000 for others; *P* < .001) (Figure, C).

Finally, we grouped drug approvals by tumor type into 2 categories: drugs approved for disease areas with more federal research funding in fiscal years 2015 to 2020 (lung, colorectal, breast and prostate cancer, and leukemias) and those approved for other disease areas (all other tumor types). There was no difference in annual cost between the 2 groups (median $191 000; IQR, $171 000-$255 000 for the more funded vs $196 000; IQR, $170 000-$307 000 for the less funded tumor types; *P* = .39).

Tumor types receiving federal funding were less likely to have drugs approved based on a novel mechanism of action (9 of 94 [9.6%] vs 20 of 130 [15%]; *P* < .001).

## Discussion

Our analysis is consistent with prior work^4,8^ and may help adjudicate a disagreement as to whether drug price is associated with innovation of anticancer drugs in the marketplace. Notably, there is no difference between the price of drugs with a novel mechanism of action and subsequent drugs based on the same mechanism, although there is wide variability in prices.

Although the first-in-class designation is often cited as the reason for high cost, the higher price of gene therapy, dominated by chimeric antigen receptor (CAR) T-cells, may be associated with the autologous nature of the drug, which makes it more dependent on the supply chain and shipment logistics, and does not benefit from the economies of scale to the extent that other treatment modalities do. Yet, even here, cost of the product is often several times that of manufacturing,^10^ which may in part be explained by the one-time nature of CAR T-cell therapy compared with chronic administration of other treatment modalities. However, there is no such explanation for the high cost of small molecules, especially when compared with biologics which have similar or, arguably, even more demanding development and manufacturing requirements. Our results reject the conclusion that cost is based on novelty alone.

One could argue that most measures of novelty we used are too granular, and that a bigger-picture perspective does point to a difference in price. The first monoclonal antibodies were approved in the 1990s and now benefit many patients with all stages of cancer at a median annual cost of $185 000, followed by tyrosine kinase inhibitors in the 2000s, the benefit of which is largely limited to metastatic tumors with specific disease-driving mutations for a median price of $239 000, and CAR T-cell therapy in the 2010s, which can help the fewest patients at the greatest cost ($448 000). This interpretation implies that innovation in cancer drug development benefits an ever-shrinking number of patients at an ever-increasing price, and discounts the important incremental improvements within each broad class of drugs.

Magnitude of benefit is another potentially rational factor to justify pricing, and value-based pricing seeks to use this concept. Drugs that bring greater improvements to the same end point or improve more objective end points (ie, progression-free survival rather than overall response rate, or overall survival rather than either) may cost more. However, evidence has shown the contrary. In a 2022 analysis,^7^ the median annual cost of drugs approved based on improvement in overall survival was the lowest of the 3 end points, indicating that factors other than novelty play an important role in drug pricing. It also found that among the drugs approved on the basis of response rate, there was a weak correlation between cost and the magnitude of that gain in response rate, and the same was true in the categories of drugs approved on the basis of gains in progression-free and overall survival.^7^

The number of new cancer drug approvals has become a quality metric for regulatory agencies,^11^ which makes it susceptible to Goodhart’s law (when a measure becomes a target, it ceases to be a good measure).^12^ The number of new drugs approved per year doubled from 51 in the 2009 to 2013 period^8^ to 119 in the 2015 to 2020 period analyzed in this study. At the same time, the median annual cost increased by more than 60% from approximately $120 000 previously reported^8^ to $195 245.

However, if the major factor associated with this price increase was the development needed to bring twice as many drugs to market, a similar trend should be found among individual manufacturers — those with more drugs approved would also have higher prices. Our analysis suggested the opposite: manufacturers with more approved drugs had lower median costs of their drugs, indicating that the relationship between price and number of drugs is not as straightforward as one might intuit by looking only at US-level data.

A 2017 analysis^5^ showed the estimated cost to bring a single cancer drug to market was $648.0 million and provided a median revenue of $1658.4 million, although other nontransparent analyses reveal much high prices — up to $4.6 billion.^13,14^ However, these costs are not borne by manufacturers alone. All stages of drug development may receive federal funding either directly through programs such as Small Business Innovation Research, or indirectly through partnerships between industry and academic institutions vying for federal grants. If direct costs of new drug development were responsible for the high prices they command once approved, drugs developed and approved for disease areas which receive less federal funding would be expected to cost more.

In our analysis, however, the cost did not vary between different tumor types with more or less funding, indicating that this component, if it exists, is negligible compared with other forces behind the price. The response of 13 heads of pharmaceutical companies to President Biden’s formulation of this question, “What the market will bear,” points to a factor much greater than cost of innovation.^15^

### Strengths and Limitations

Our study has several strength and limitations. One strength is the lengthy and recent period under study, which allowed us to update previous findings in this area of research. Second, we used a robust classification of novelty of mechanism of action encompassing all tumor type and within specific tumor types. Third, we analyzed previously unexplored associations between manufacturer productivity, estimates of federal research funding, and ultimate cancer drug price.

The first limitation may be that we collected the manufacturer data listed on the FDA label—which is often neither the entity that developed the drug nor reflective of available rebates—under the assumption that the R&D cost would be passed on to the manufacturer by way of licensing fees or the acquisition price, and ultimately reflected in the drug’s final price. Second, we did not account for the R&D costs of drugs that never reached the market and the capacity of drug manufacturers to absorb those costs without affecting the price of their approved drugs. Third, we used only the budget of the National Cancer Institute as a surrogate for all federal funding, which also includes the National Science Foundation and Department of Defense, among others.

## Conclusions

In this cross-sectional study, we found that despite increases in recent cancer drug approvals, drug pricing cannot be explained by innovations alone. Specifically, the novelty of a drug is not associated with pricing. In concordance with others, we did not find that industry expenditure on R&D explains the US costs of cancer drugs.

## Notes

Supplement 1. **eTable.** Distinct Drugs Approved by the US Food and Drug Administration (2015-2020), by Novelty Across all Tumors

Supplement 2.
Data Sharing Statement
