# Supplementary material for: Cancer Drug Price and Novelty in Mechanism of Action
Source: JAMA Netw Open. 2023 Dec 11;6(12):e2347006. doi: 10.1001/jamanetworkopen.2023.47006 (PMC10714245; doi:10.1001/jamanetworkopen.2023.47006)
Supplement: Supplement 1. — eTable. Distinct Drugs Approved by the US Food and Drug Administration (2015-2020), by Novelty Across all Tumors [file jamanetwopen-e2347006-s001.pdf]

Supplemental Online Content

Miljković MD, Tuia J, Olivier T, Haslam A, Prasad V. Association between cancer drug price and novelty in mechanism of action. *JAMA Netw Open.* 2023;6(12):e2347006. doi:10.1001/jamanetworkopen.2023.47006

eTable. Distinct Drugs Approved by the US Food and Drug Administration (2015-2020), by Novelty Across all Tumors

This supplemental material has been provided by the authors to give readers additional information about their work.

© 2023 Miljković MD et al. *JAMA Network Open*.

eTable. Distinct Drugs Approved by the US Food and Drug Administration (2015-2020), by Novelty Across all Tumors

	Approved based on a new mechanism of action	First approvals of a next-in-class drug	Subsequent approvals of the same drug
Distinct drugs, n	29	57	59
Distinct drugs with subsequent approvals	9	17	33

© 2023 Miljković MD et al. *JAMA Network Open*.
