# Supplementary material for: Cancer Drug Price and Novelty in Mechanism of Action
Source: JAMA Netw Open. 2023 Dec 11;6(12):e2347006. doi: 10.1001/jamanetworkopen.2023.47006 (PMC10714245; doi:10.1001/jamanetworkopen.2023.47006)
Supplement: Supplement 2. — Data Sharing Statement [file jamanetwopen-e2347006-s002.pdf]

Data Sharing Statement

Miljković. Cancer Drug Price and Novelty in Mechanism of Action. *JAMA Netw Open*.
Published December 11, 2023. doi:10.1001/jamanetworkopen.2023.47006

Data
Data available: No
